# A watch-summarize-question gamified learning approach for oral health: learning achievement, motivation, and flossing skills

**DOI:** 10.1186/s12903-025-06888-1

**Published:** 2025-10-08

**Authors:** Ching-Yi Chang, Ming-Ching Wu, Jie Chi Yang

**Affiliations:** 1 School of Nursing, College of Nursing, Taipei Medical University, Taipei, Taiwan; 2 Graduate Institute of Network Learning Technology, National Central University, Taoyuan, Taiwan; 3 Research Center for Science and Technology for Learning, National Central University, Taoyuan, Taiwan

**Keywords:** Flossing skills, Gamification, Learning motivation, Watch-summarize-question

## Abstract

**Background:**

Flossing skills are critical to promoting oral health. While various technologies have been employed in previous research to teach flossing skills, their effectiveness remains unclear. To address this gap, this study proposed a Watch-Summarize-Question (WSQ) gamified learning approach to enhance oral health education with a focus on flossing skills. The study investigated the approach’s impact on learning achievement, learning motivation, and flossing skills performance.

**Methods:**

A quasi-experimental study was adopted, with the experimental group using the WSQ gamified learning approach and the control group using a video-based learning method.

**Results:**

The results revealed that the WSQ gamified learning approach significantly improved learning achievement in flossing knowledge compared with the video-based learning method. However, both groups demonstrated comparable effects on learning motivation and flossing skills performance.

**Conclusion:**

These findings indicate that the WSQ gamified learning approach is a promising strategy for oral health promotion. Educators are encouraged to integrate appropriate technologies and learning strategies to effectively enhance learners’ flossing skills.

## Introduction

Oral health is a critical component of overall health [1]. Maintaining good oral health helps prevent dental and gum diseases, such as cavities, periodontal disease, and oral infections, which, if left untreated, can lead to more serious health issues [2]. Research has shown that poor oral health is associated with various systemic diseases, including cardiovascular disease, diabetes, and respiratory diseases [3]. By promoting oral health, individuals can reduce the risk of these systemic conditions. Oral health encompasses not only the health of teeth and gums but also plays a vital role in overall health and quality of life. Regular dental check-ups and proper oral hygiene practices are essential for maintaining oral health [4]. Flossing, for example, effectively cleans the contact areas between teeth and gums, preventing gum inflammation and cavities, and protecting overall dental health [5]. Using dental floss complements brushing by providing a more thorough oral cleaning, enhancing oral hygiene, and improving quality of life.

Given the importance of flossing, various technologies have been integrated into teaching flossing techniques. For example, Özişçi utilized instructional videos and animations on platforms like YouTube and other video-sharing sites to teach proper flossing skills [6]. Additionally, Huang et al. and Lin et al. investigated the application of virtual reality (VR) and augmented reality (AR) tools in oral care [7, 8]. VR environments allow learners to practice flossing techniques virtually, while AR applications overlay instructional information in real-world settings, offering real-time guidance on proper flossing techniques. However, teaching flossing presents unique challenges due to its reliance on motor skills, engagement, and feedback. To address these challenges, more integrative and engaging strategies, such as gamification, are essential to enhance knowledge and skill acquisition in flossing techniques.

Gonçalves et al. characterize gamification as an approach that incorporates game elements into educational content through an engaging digital platform to support the development of knowledge and abilities [9]. By offering an engaging and interactive experience, gamification motivates learners to actively participate in the learning process [10]. It has evolved into a valuable approach to education, delivering content in an enjoyable and effective manner [11]. Additionally, gamification emphasizes voluntary participation, which is essential for fostering multiplayer engagement in the learning process [12].

Although previous research highlights the benefits of gamification, its impact on learning can be diminished if instructional strategies are not properly integrated. To address this, the current study adopts the Watch-Summarize-Question (WSQ) learning strategy, a three-stage learning activity where learners watch instructional videos, summarize key points, and pose questions to deepen understanding during the learning process [13]. The WSQ strategy has shown significant effectiveness in various educational contexts. For example, Karabacak et al. reported that the WSQ approach offers structured support that significantly enhances students’ clinical nursing abilities [14]. Similarly, Lin et al. demonstrated that it not only improves practical abilities in upper secondary education but also encourages learners to reflect on their learning experiences [15].

Despite extensive research on gamified learning, much of the focus has been on engagement, motivation, and knowledge acquisition, with limited exploration of integrating the WSQ strategy. While the combination of gamification and the WSQ strategy has been studied in nursing education, its application in oral health education, particularly in teaching flossing skills, remains underexplored. To address these gaps, this study proposes a WSQ gamified learning approach to facilitate the acquisition of flossing skills. The research questions guiding this study are:

1. Is there a significant difference in learning achievement of flossing knowledge between participants using the WSQ gamified learning approach and those using a video-based learning method?
2. Is there a significant difference in learning motivation between participants using the WSQ gamified learning approach and those using a video-based learning method?
3. Is there a significant difference in flossing skills performance between participants using the WSQ gamified learning approach and those using a video-based learning method?

## Literature review

### Flossing skills

Flossing is a crucial component of oral hygiene, essential for preventing gum diseases and maintaining overall dental health. By effectively removing plaque and food debris from the gum line and between teeth, flossing addresses the primary causes of gingivitis and periodontal disease [16]. Without regular flossing, plaque can accumulate, leading to swollen, bleeding gums, and even loose teeth. Although brushing removes most plaque, it cannot reach the narrow spaces between teeth. Flossing complements brushing by cleaning these hard-to-reach areas, thereby preventing the development of cavities [17]. Additionally, flossing contributes to maintaining fresh breath by eliminating food particles and plaque that can break down and produce unpleasant odors. Thus, flossing plays an essential role in ensuring both oral health and fresh breath. Good oral hygiene habits extend beyond the health of teeth and gums, carrying significant implications for overall health. Research indicates a strong connection between oral health and systemic conditions such as cardiovascular diseases and diabetes [18]. Regular flossing, alongside other oral hygiene practices, not only helps prevent dental and gum diseases but also reduces the frequency of dental visits and associated treatment costs.

In the era of digital technology, innovative tools have been introduced to enhance oral health education and encourage better oral hygiene habits. Many dentists and oral hygiene experts now share instructional videos on platforms like YouTube, demonstrating proper flossing techniques [6]. Animated demonstrations are particularly effective for teaching children and visual learners, offering an intuitive way to grasp correct flossing techniques [19]. Mobile oral care applications also play a significant role by providing oral hygiene guides, instructional videos, and reminder functions to help users establish regular flossing habits [20]. These interactive apps simulate the flossing process and offer real-time feedback and suggestions. Online courses and webinars further enhance flossing education by allowing participants to interact with experts, ask questions, and receive personalized advice [21]. Social media platforms serve as another avenue for sharing flossing techniques through videos, pictorial guides, and community discussions [22]. By joining online oral care communities, users can exchange experiences and advice, fostering motivation and accountability for maintaining regular flossing habits.

These technological approaches not only make learning about flossing more convenient and engaging but also raise awareness about the importance of oral hygiene. They promote the development of lifelong oral care habits, which are critical for both oral and systemic health. While traditional educational methods like instructional videos are valuable, emerging technologies such as gamification offer promising opportunities to address existing challenges and make flossing education more effective and engaging.

### Gamification

Deterding et al. define gamification as “the use of game design elements in non-game contexts [23].” This concept leverages the engaging and interactive nature of games to enhance learning experiences, making them more enjoyable and effective [24]. The application of gamification in education has become increasingly prevalent, positioning it as a significant topic in educational research [25]. Gamification incorporates game-like elements such as challenges, feedback, achievements, and competition to stimulate learners’ interest and persistence. The theoretical foundation of gamification is primarily rooted in self-determination theory [26], which emphasizes the role of intrinsic motivation in learning. By offering immediate feedback, clear goals, and meaningful challenges, gamification fulfills learners’ needs for autonomy, competence, and relatedness, thus enhancing intrinsic motivation.

Gamification employs features like points, badges, leaderboards, and challenges to stimulate interest and engagement. Khaledi et al. stated that this approach significantly enhances motivation and participation by creating interactive learning environments where learners can internalize content through simulations and role-playing [27]. Mims et al. emphasized that prompt responses and engaging tasks within gamified learning assist students in enhancing motivation and confidence, and reinforcing acquired knowledge [28]. Gamified learning environments are inherently engaging, fostering participation and interest. By providing immediate feedback, gamification helps students monitor their progress and identify areas for improvement, enabling them to adjust their learning strategies. Additionally, gamification often reduces stress and anxiety, creating a more motivating and enjoyable educational experience [29].

The impact of gamification spans various disciplines. For example, Nik Fauzi et al. illustrated its value in language learning by strengthening students’ linguistic abilities through immersive simulations and hands-on interaction [30]. da Silva Júnior et al. indicated that gamified approaches improve the understanding of intricate topics in science instruction, especially within the context of chemistry laboratory activities [31]. Similarly, Topalli et al. discovered that the use of gamified methods enhances educational achievement in medical training, particularly in domains that demand advanced expertise and proficiency, such as operative techniques and clinical judgment [32].

Previous studies also highlight the increasing adoption of gamified interventions and serious game-based approaches in dental education. For example, Krause et al. developed and evaluated a serious game to strengthen dental students’ factual knowledge, finding that the competitive gameplay context enhanced both knowledge retention and learner engagement [33]. Similarly, Khorasanchi et al. designed a serious game on laser applications in dentistry, reporting high levels of student satisfaction with the interactive gamified learning environment [34]. Mendonça et al. created a serious game for children’s oral health education using a user-centered design approach, demonstrating its potential to promote preventive knowledge about dental caries through enjoyable gameplay [35]. More recently, Badr and Khalifa employed a virtual escape room to improve undergraduate dental students’ understanding of differential diagnosis in oral radiology, which resulted in significant knowledge gains [36]. Another study by Wu et al. implemented an educational board game to train dental and dental hygiene students on patient safety, showing the value of gamification in enhancing both knowledge and awareness of patient safety issues [37].

While these studies demonstrate the potential of gamification in dental education, most have not embedded effective learning strategies within their design to maximize educational outcomes. In contrast, our approach uniquely integrates the WSQ strategy with gamification elements, emphasizing scaffolded learning sequences, learner reflection, active engagement, and the application of knowledge, thereby extending the pedagogical value of gamification in dental education.

## WSQ learning strategy

The WSQ learning strategy, proposed by Kirch, guides students to summarize key points and pose questions while watching videos, facilitating their self-directed learning process [13]. This strategy aims to enhance student engagement and learning outcomes by emphasizing active participation and promoting deeper learning. The WSQ strategy consists of three stages: (1) Watch: Students begin by watching video materials, typically provided by the teacher as part of the course. This stage requires focused attention to understand key concepts and messages within the video content. (2) Summarize: After watching the video, students summarize the main points. This stage helps organize information, deepen understanding, and internalize the knowledge acquired. (3) Question: In the final stage, students generate questions based on the video content. These questions can clarify unclear concepts or explore topics of interest for further investigation. This stage encourages critical thinking and prepares students for discussions with peers and teachers during subsequent lessons. By integrating video content with summarization and question-posing, the WSQ strategy supports knowledge absorption and the development of essential skills, such as information organization, critical analysis, and problem-solving [15].

Research has demonstrated the WSQ strategy’s effectiveness in enhancing learning outcomes. For example, Hsia et al. found that it provides a learning scaffold that promotes reflection and critical thinking while motivating students to engage more deeply with the content [38]. Similarly, Liu et al. highlighted the benefits of combining the WSQ strategy with peer assessment, which improved students’ learning performance and fostered communication among peers [39]. However, the study noted that the WSQ strategy had a limited impact on improving students’ learning attitudes, likely due to factors such as personal interest, intrinsic motivation, and course design. Additionally, Chang et al. demonstrated that integrating gamified learning with the WSQ strategy positively impacted nursing students’ training in suctioning skills [40]. This combination utilized the interactivity and appeal of gamification alongside the deep learning mechanisms of the WSQ strategy, creating an engaging and educationally meaningful environment. This approach is particularly valuable in nursing education, where students must develop both theoretical knowledge and practical skills to succeed in their future careers.

These studies highlight the WSQ strategy as an effective scaffold that provides support and guidance during the learning process, thereby enhancing learning outcomes. However, its application in oral health education, particularly in teaching flossing skills, remains unexplored. To address this gap, this study combines gamified learning with the WSQ strategy to design practical, engaging activities for teaching flossing skills.

## Methods

### Research design

A quasi-experimental study was conducted to assess learning achievement, learning motivation, and flossing skills performance. The study included two groups: an experimental group that participated in a WSQ gamified learning approach as part of their health promotion activity, and a control group that received instruction through a video-based learning method.

### WSQ gamified learning system

**Fig. 1:**
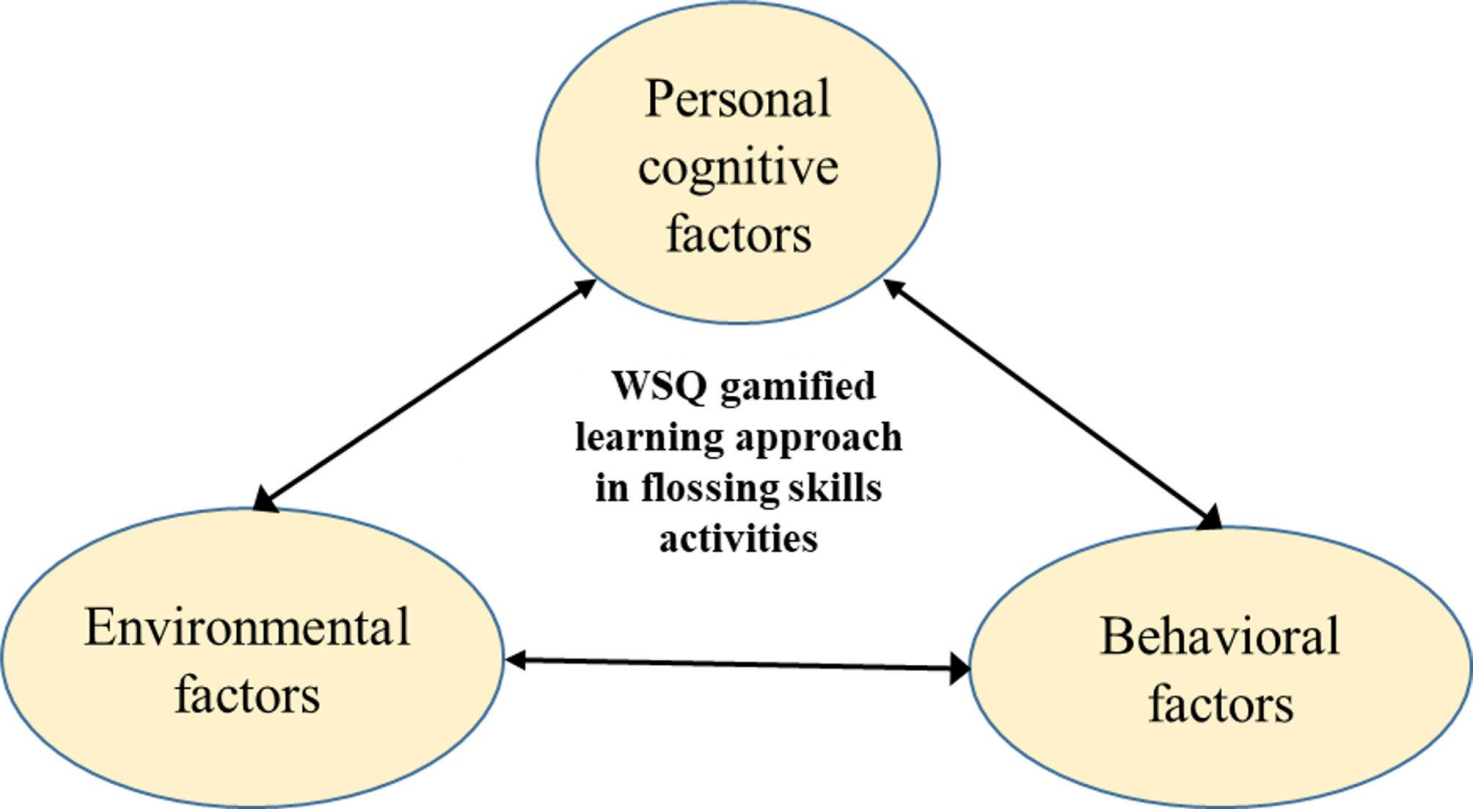
WSQ gamified learning approach in flossing skills activities incorporating SCT

The WSQ gamified learning system integrates the WSQ strategy with an online gamified platform *Gather* (https://www.gather.town) [41]. The system incorporates Social Cognitive Theory (SCT) [42], which consists of three key factors: personal cognitive, environmental, and behavioral factors. The WSQ gamified learning approach in flossing skills activities incorporating SCT is illustrated in Fig. 1, emphasizing learners’ learning processes within the social context of a gamified learning approach. The three factors include: (1) Personal cognitive factors: Learners’ beliefs, attitudes, expectations, abilities, and past experiences with using dental floss to promote oral health; (2) Environmental factors: External influences such as the *Gather* learning environment, social context, physical setting, and cultural context; and (3) Behavioral factors: Learners’ concrete actions in using dental floss, including their confidence in successfully performing specific flossing tasks. This confidence influences their choice of oral health-promoting activities, effort investment, and persistence in facing challenges in flossing for oral hygiene.

The WSQ gamified learning system was developed by incorporating several interface design principles adapted from [43], including sequential WSQ flow, freedom to fail, rapid feedback and rewards, progression visuals, storytelling and context, thoughtful gamification mechanics, inclusive design, consistent user interface (UI), and interaction, as summarized in Table 1. In addition, the system architecture was reviewed by two educational experts, yielding an Expert Validity Index (EVI) of 0.85, indicating a high level of expert agreement on its design validity.

**Table 1:** Interface design principles for the WSQ gamified learning system

Principle	Description
Sequential WSQ flow	Clear progression through Watch → Summarize → Question
Freedom to fail	Encourage re-engagement and reduce perceived stakes
Rapid feedback and rewards	Provide instant validation and gamification elements (e.g., feedback, points)
Progression visuals	Use milestone bars and unlockable content to track learning progress
Storytelling and context	Embed WSQ tasks within a meaningful narrative
Thoughtful gamification mechanics	Apply effective dynamics, mechanics, and components
Inclusive design	Support diverse learners and avoid overstimulation
Consistent UI	Maintain a uniform design and layout across stages
Interaction	Integrate interactive tools to deepen learning

The three stages of the WSQ strategy are outlined in Table 2. By applying SCT in the WSQ gamified learning system, learners are guided through observational learning, self-regulation, reflective thinking, active learning, and reinforcement, all within an interactive and engaging gamified learning environment. This approach facilitates the acquisition of oral health knowledge and flossing skills.

**Table 2:** The three stages of the WSQ gamified learning approach incorporating SCT

Stage	Description
Watch	Observational Learning: Learners acquire knowledge and skills by observing others. In this stage, learners watch educational videos or interactive demonstrations integrated within the gamified learning system.
	Modeling: The content showcase expert models or peer demonstrations, displaying essential skills and strategies to solve challenges in the gamified learning system.
	Interactive Feedback: Learners can replay sections or slow down videos to carefully observe flossing techniques. This functionality reinforces learning by encouraging focused attention on modeled behaviors.
	Peer Influence: Integrating peer avatars or collaborative group activities fosters a gamified learning environment, enabling learners to observe and adopt effective strategies through peers.
Summarize	Self-Regulation: Summarizing encourages learners to practice self-regulation by taking control of their learning and reflecting on what they have learned.
	Goal Setting: The gamified learning system encourages learners to set specific goals for their summaries, promoting active engagement and better comprehension.
	Reflective Thinking: Built-in prompts guide learners to reflect on why certain actions or strategies were effective, deepening their understanding.
	Self-Efficacy: Successfully summarizing complex concepts enhances learners’ confidence. With adaptive feedback, the gamified learning system fosters self-efficacy by helping learners recognize their capacity to understand and articulate new knowledge.
Question	Active Learning through Questioning: In this stage, learners actively generate questions based on their acquired knowledge, fostering deeper engagement with the content.
	Reinforcement and Rewards: A reward system promotes the development of meaningful questions. The gamified learning system provides points for insightful questions, motivating learners to engage critically with the content.
	Peer and Instructor Interaction: Learners share their questions with peers or instructors, facilitating knowledge reinforcement through social interaction and feedback.
	Problem-Solving: Creating and answering questions boosts problem-solving skills. Through feedback on their questions and visible progress, learners build confidence in their reasoning skills.

Through the WSQ gamified learning approach, learning activities are structured into three stages:

1. Watch Stage: Learners watch instructional videos embedded in the system that demonstrate flossing techniques. The content is segmented according to different teeth to simulate a complete oral care routine, supporting targeted knowledge acquisition. Learners may pause, rewind, or replay the videos as needed to strengthen their comprehension.
2. Summarize stage: Learners summarize key points from the videos they watched and record them simultaneously in the interactive chatroom. Each summary is represented through a series of flossing skill scenarios, simulating real-life flossing situations. After each flossing step, learners reflect on their performance, identifying strengths and areas for improvement. This process is supported by the “Flossing Warrior,” who helps learners evaluate and clarify their flossing skills and deepen their understanding of the content.
3. Question stage: Learners pose their questions in the chatroom and engage in discussions with the “Flossing Warrior” to brainstorm solutions. As learners contribute more questions, they unlock more advanced flossing skills, progressing from basic flossing techniques to comprehensive oral hygiene management tasks.

**Fig. 2:**
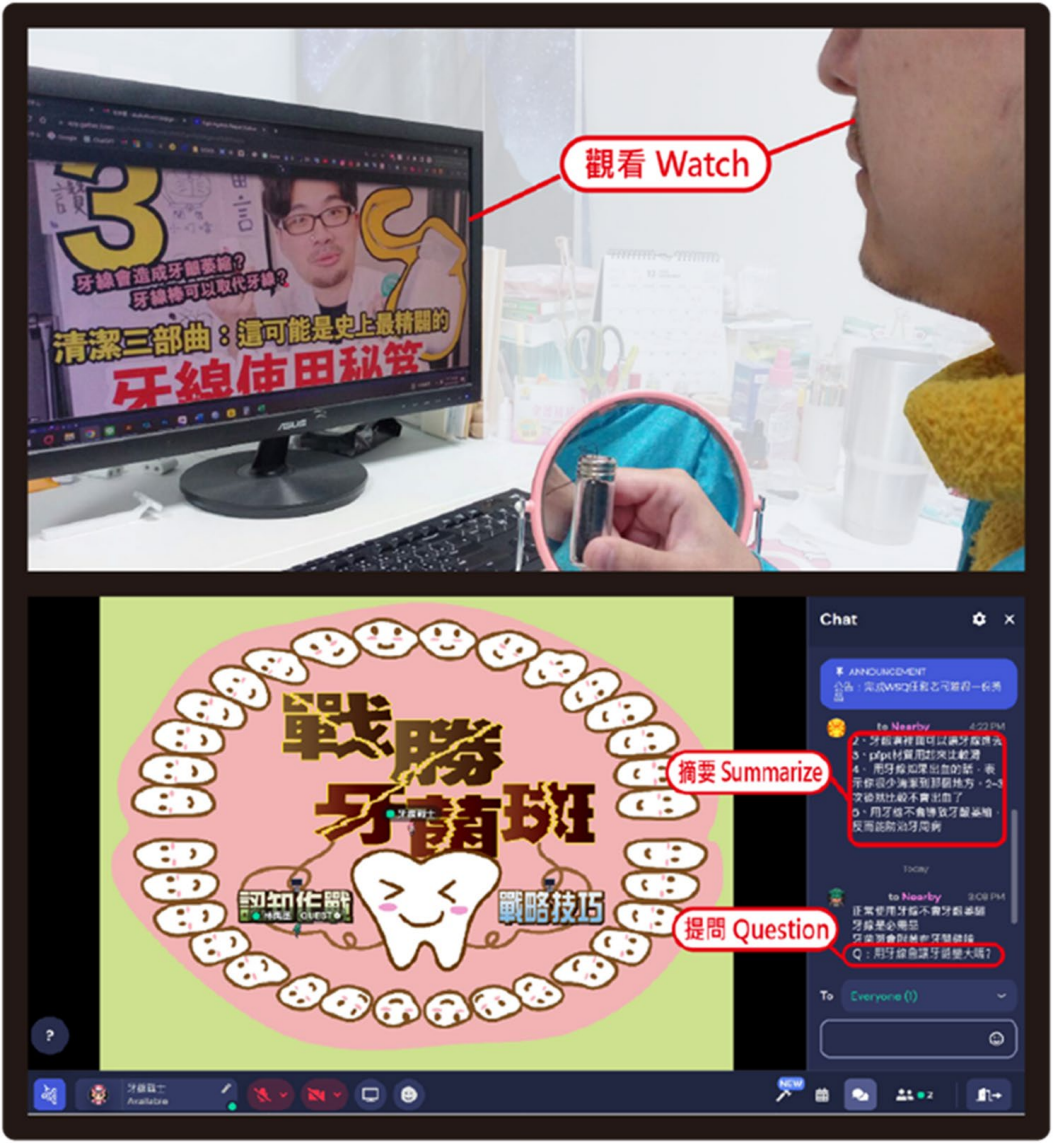
Screenshots of the three WSQ stages in the gamified learning system

Across the three stages, feedback is provided by the “Flossing Warrior,” a facilitator represented by an avatar in the virtual environment and enacted by the instructor or trained peer mentor. The facilitator monitors learners’ performance, responds to questions in real time, and delivers adaptive guidance based on learners’ inputs during the Summarize and Question stages. Fig. 2 illustrates screenshots of the three stages in the WSQ process.

The WSQ gamified learning system functions as a role-play learning environment where learners interact through personalized avatars. Gamification elements integrated into the system include point-based rewards, progress indicators, time-limited missions, and skill unlocks. Point-based rewards are granted for attentive video watching, meaningful summaries, and insightful questions. Points accumulate across the three WSQ stages and are displayed as motivational feedback, reinforcing self-paced mastery without fostering competition. Progress indicators, such as progress bars and checklists, enable learners to monitor their advancement through the WSQ stages. Each completed task—such as watching a video, summarizing content, or asking a question—triggers a visual update that reinforces their sense of accomplishment. These indicators also track the completion of flossing-related learning units, thereby encouraging structured and goal-driven engagement. Time-limited missions require learners to complete specific tasks within set timeframes—for example, identifying key flossing techniques while watching a video module or generating summaries and questions within a designated window. These constraints simulate real-world decision-making contexts and sustain momentum in the learning process.

Skill unlocks further scaffold progression: learners begin with fundamental content (e.g., front teeth flossing) and, by meeting performance criteria such as accurate summaries or high-level questions, gain access to more complex scenarios (e.g., back molar areas, gumline care, and interdental device usage). Unlocks are highlighted through visual effects and notifications, reinforcing achievement while ensuring learning builds progressively on prior mastery. In sum, the incorporation of SCT and gamification elements within the three WSQ stages ensures that the WSQ gamified learning approach is both pedagogically grounded and dynamically operationalized. By engaging learners in structured, interactive, and measurable activities, the WSQ gamified learning system supports the practice and mastery of flossing techniques while fostering active participation, skill development, and long-term oral health management.

### Instructional content

The instructional content used in this study consisted of two dental flossing tutorial videos developed by two oral health experts. The first video (25 min) introduced the importance of flossing and provided foundational knowledge, covering topics such as why flossing is necessary, how to choose the right floss, common flossing mistakes, precautions when purchasing floss, whether flossing can cause gum recession, and whether floss picks can replace floss. The second video (20 min) focused on proper flossing skills to promote oral health. It demonstrated each step in detail, including selecting the appropriate floss, measuring the correct length, wrapping it around the fingers, and sliding it up and down between teeth. The video also highlighted common errors that may cause gum injury or other oral problems and explained how to avoid them.

Both the experimental and control groups viewed the same videos but through different approaches: the experimental group engaged with the videos guided by the WSQ gamified learning approach, whereas the control group watched them directly without additional support. Table 3 presents the differences between the two groups in terms of instruction content, intervention time, and learning approach.

**Table 3:** Differences between the experimental and control groups

	Experimental group	Control group
Instructional content	Two dental flossing tutorial videos	The same two dental flossing tutorial videos as the experimental group.
Intervention time	70 min	70 min
Learning approach	Participants watched the videos guided by the WSQ gamified learning approach. They were required to complete the three stages of WSQ activities (Watch, Summarize, Question) with gamified elements such as point-based rewards, progress indicators, time-limited missions, and skill unlocks.	Participants used a traditional video-based learning method, watching the videos directly without additional support. They were permitted to rewatch the videos as needed.

### Participants

To assess the effectiveness of the WSQ gamified learning approach, 48 participants (24 males and 24 females) were recruited through a university’s website. All participants voluntarily agreed to participate in the experiment and signed an informed consent form. Participants were aged between 20 and 24 years, with an average age of 22. They were randomly divided into two groups: an experimental group that implemented the WSQ gamified learning approach (*N* = 24), and a control group that utilized the video-based learning method (*N* = 24).

### Experimental procedure

The experimental procedure consisted of four sessions with a total duration of 130 min. In the first session (20 min), participants completed a pretest assessing their flossing skills knowledge and a pre-questionnaire evaluating their learning motivation. The second session (20 min) provided a brief instruction on basic knowledge of oral health, covering plaque prevention, proper flossing techniques, and fundamental oral health concepts. The third session (70 min) served as the main intervention, during which the experimental group learned through the WSQ gamified learning approach, while the control group used a video-based learning method. In the fourth session (20 min), participants completed a posttest on flossing skills knowledge and a post-questionnaire on learning motivation, followed by a practical flossing activity that was recorded and later evaluated by two oral health experts.

### Measurement tools

The measurement tools used in this study included a flossing skills knowledge test, a learning motivation questionnaire, and a flossing skills evaluation rubric. To measure learning achievement, a flossing skills knowledge test was developed based on the American Dental Association’s guidelines on flossing (https://www.mouthhealthy.org/all-topics-a-z/flossing). The test composed of 20 questions, each worth 5 points, for a total possible score of 100 points. According to Bloom’s Taxonomy, 12 questions targeted lower-order cognitive skills, such as remembering and understanding (e.g., identifying the correct flossing sequence and recognizing appropriate flossing tools), while 8 questions assessed higher-order thinking skills, including applying, analyzing, and evaluating (e.g., determining the most effective flossing method for specific dental conditions, analyzing potential causes of ineffective flossing, and evaluating alternative flossing techniques). The test items were reviewed by two oral health experts to ensure content validity. The internal consistency reliability of the flossing skills knowledge test, measured by the KR-20 coefficient, was 0.82, indicating good reliability for assessments with dichotomously scored items.

Learning motivation was assessed using a questionnaire by Li and Keller [44]. The questionnaire measured four dimensions: attention, relevance, confidence, and satisfaction, with each dimension containing nine items, totaling 36 items. To encourage careful responses, eight of these items were negatively worded. Responses were measured using a five-point Likert scale, where “1” indicated strong disagreement and “5” indicated strong agreement. The Cronbach’s alpha value for the questionnaire was 0.96, indicating strong reliability.

Flossing skills performance was evaluated using a rubric developed based on the “5 steps for flawless flossing” from the American Dental Association (https://www.mouthhealthy.org/all-topics-a-z/flossing). The rubric included five items corresponding to the five flossing steps, each assigned a maximum score of 20 points, resulting in a total possible score of 100 points. Participants’ flossing skills performance was rated by two oral health experts using a three-point scale: 0 points for “completely inaccurate,” 10 points for “roughly accurate but needs improvement,” and 20 points for “very accurate.” Inter-rater reliability, measured using Cohen’s kappa, was 0.85, indicating a high level of agreement.

## Results

### Learning achievement

Before the experiment, both groups completed a pretest to evaluate their initial knowledge in flossing. The experimental group had a mean score of 77.92 (SD = 11.51), while the control group had a mean score of 77.29 (SD = 12.07). An independent samples *t*-test revealed no significant difference between the two groups (*t* = 0.184, *p* >.05), indicating that the two groups had similar flossing skills knowledge prior to the experiment. Subsequently, ANCOVA was employed to compare the posttest scores between the two groups while controlling for the influence of their pretest scores. In the ANCOVA analysis, pretest scores served as the covariate, and posttest scores were treated as the dependent variable. The assumptions of homogeneity of regression coefficients across the groups was tested and showed that the *F* value did not violate the homogeneity assumption (*p* >.05). The ANCOVA results (Table 4) revealed a significant difference in posttest scores between the two groups (*F* = 11.10, *p* <.01). The adjusted mean score for the experimental group was 99.13 (standard error = 0.83), compared with 95.24 (standard error = 0.83) for the control group. These findings suggest that the WSQ gamified learning approach implemented with the experimental group significantly enhanced participants’ learning achievement in flossing knowledge compared with the control group, which utilized a video-based learning method.

**Table 4:** ANCOVA results of learning achievement

Group	*N*	Mean	SD	Adjusted mean	Std. error	F	η^2^
Experimental group	24	99.17	1.90	99.13	0.83	11.10^**^	0.198
Control group	24	95.21	5.61	95.24	0.83		

^**p<.01^

### Learning motivation

Before the experiment, both groups completed a pre-questionnaire to evaluate their initial learning motivation. The experimental group had a mean score of 3.54 (SD = 0.55), while the control group had a mean score of 3.63 (SD = 0.49). An independent samples *t*-test revealed no significant difference between the two groups (*t* = −0.56, *p* >.05), indicating that the two groups had similar learning motivation prior to the experiment. Subsequently, ANCOVA was employed to compare the post-questionnaire scores of learning motivation between the two groups while controlling for the influence of their pre-questionnaire scores. The assumptions of homogeneity of regression coefficients across the groups was tested and showed that the *F* value did not violate the homogeneity assumption (*p* >.05). The ANCOVA results (Table 5) revealed no significant difference in post-questionnaire scores of learning motivation between the two groups (*F* = 0.087, *p* >.05). The adjusted mean score for the experimental group was 4.07 (standard error = 0.07), compared with 4.04 (standard error = 0.07) for the control group. These findings suggest that the WSQ gamified learning approach had a comparable effect on participants’ learning motivation when compared with the video-based learning method.

**Table 5:** ANCOVA results of learning motivation

Group	*N*	Mean	SD	Adjusted mean	Std. error	F	η^2^
Experimental group	24	4.06	0.33	4.07	0.07	0.087	0.002
Control group	24	4.06	0.42	4.04	0.07		

### Flossing skills performance

An independent samples *t*-test was conducted to compare the flossing skills performance between the two groups after the experiment. The results revealed no significant difference between the groups (*t* = 1.31, *p* >.05) (Table 6). However, the mean score of the experimental group (Mean = 73.96, SD = 11.23) was slightly higher than the control group (Mean = 69.38, SD = 12.95). These findings suggest that although the difference between the two groups was not significant statistically, the WSQ gamified learning approach resulted in slightly higher scores in flossing skills performance compared with the video-based learning method.

**Table 6:** t-test results of flossing skills performance

Group	*N*	Mean	SD	t
Experimental group	24	73.96	11.23	1.31
Control group	24	69.38	12.95	

## Discussion

The results indicated that the WSQ gamified learning approach shows significant potential to improve participants’ learning achievement in flossing knowledge. The WSQ gamified learning approach, which allows participants to browse and search learning content, not only aids in effectively organizing knowledge but also assists participants in reorganizing their understanding of flossing knowledge. This demonstrates that the WSQ gamified learning approach facilitates deep reflection and helps participants expand their learning scope while reorganizing acquired knowledge. These findings align with Chang et al., who posited that gamified learning environments enable learners to actively engage with content through interactive tasks [40]. Such hands-on engagement promotes better understanding and retention of proper flossing techniques compared with passively watching videos. Real-time feedback within the gamified learning system helps learners correct errors and refine their techniques, fostering memory and practical application. Furthermore, learners often report higher satisfaction with this interactive learning approach than with passive instructional videos. Previous research on gamified learning in health education suggests that interactive, scenario-based environments are effective in teaching procedural and motor skills [45]. For example, studies on hygiene practices and physical activities have shown that gamified learning enhances both knowledge acquisition and skill performance by incorporating repetitive practice in an engaging format [46]. This aligns with the study’s findings that gamification improves learning achievement, particularly for tasks requiring consistent practice, such as flossing. Moreover, the WSQ gamified learning approach provides tailored feedback, enabling learners to identify specific areas for improvement [15]. Unlike static video demonstrations, the system’s interactivity ensures that learners receive immediate corrections and adapt their techniques accordingly. Gamified rewards and progress tracking further motivate continuous effort and repeated practice [47], which are essential for mastering flossing skills. The success of the WSQ gamified learning approach underscores its potential in broader oral health education. Interactive gamified learning system can make oral hygiene education more engaging and effective. In contrast, the control group, which acquired knowledge through instructional videos, experienced a passive activity that limited opportunities for active practice and feedback. Learners in the control group could not confirm whether their flossing techniques were correct, potentially reinforcing improper habits. Without active participation, learners were more likely to forget the steps or nuances of proper flossing.

The results also revealed that the experimental group using the WSQ gamified learning approach showed no significant differences compared with the control group using the video-based learning method in motivation for learning flossing techniques. This indicates that the WSQ gamified learning approach does not substantially affect learners’ motivation. This finding is consistent with Hsia et al., which reported no significant differences in students’ learning motivation with the use of the WSQ strategy [38]. One possible explanation for this result lies in the design of the WSQ gamified learning approach. The current system awards points to learners for correctly answering questions but lacks diversified gamified elements such as badges and leaderboards. This design might lack sufficient challenges or rewards, diminishing learners’ sense of accomplishment or engagement [48]. Effective gamified learning requires a balance of challenge and achievable rewards. Without this balance, the system may not fully engage learners or positively impact their motivation. Additionally, learners’ familiarity with gameplay might influence motivation. For participants unfamiliar with gamified systems, engagement with the gamified learning system could be hindered, reducing its motivational potential [49]. Furthermore, individual differences in learning preferences may have contributed to the insignificant results. Some learners may prefer traditional methods or may not enjoy the interactive nature of gamified learning. If participants do not find the gamified system engaging, it would not have had a positive impact on their learning motivation. Addressing these factors could improve the WSQ gamified learning approach’s effectiveness in enhancing motivation.

Regarding flossing skills performance, the results showed that while the experimental group performed slightly better than the control group, the difference was not significant statistically. This may be attributed to the nature of flossing skills, which require repeated practice over time. The short-term experimental session may have been insufficient to produce immediate changes in learners’ flossing skills performance. As Jung et al. noted, flossing is a motor skill that necessitates hands-on practice and cannot be effectively taught or reinforced over a brief period [50]. While gamified learning enhances engagement, it may prioritize entertainment elements over the repetitive practice necessary for skill acquisition. Additionally, evidence shows that gamified learning may introduce cognitive overload, making it harder for learners to focus specifically on flossing techniques [51]. The WSQ gamified learning approach may be more effective at improving flossing knowledge rather than the hands-on skill, which may require different teaching strategies [52].

Although previous studies in dental education involving gamification or serious game-based approaches have demonstrated improvements in knowledge and engagement [33, 35–37, 44], most relied primarily on quiz-based challenges. In contrast, the WSQ gamified learning approach embeds structured self-questioning and reflective practice, incorporating metacognitive prompts that encourage deeper reflection and active reorganization of learning content, moving beyond simple quiz-based reinforcement. Unlike other gamified interventions that focus mainly on rewards or competition (e.g., badge systems or leaderboards), the WSQ gamified learning approach emphasizes learner autonomy and content exploration, aligning with constructivist principles that view learning as an active, self-directed process. Additionally, the system is underpinned by SCT, as learners engage in observational learning through instructional videos, receive guided feedback from the facilitator, and build self-efficacy through scaffolded tasks and progressive skill unlocks.

This study extends existing research by positioning the WSQ gamified learning system not merely as a motivational tool, but as a theoretically grounded pedagogical framework that scaffolds critical thinking and reflective engagement in dental education. It advances the field by demonstrating how the deliberate integration of the WSQ learning strategy with gamification can produce meaningful learning outcomes, particularly for procedural topics such as flossing.

## Conclusions

The study proposed a WSQ gamified learning approach for oral health education focused on flossing skills, comparing it with a video-based learning method. The findings demonstrated that the WSQ gamified learning approach enhanced learning achievement by enabling learners to better organize and reflect on flossing knowledge. However, no significant differences in learning motivation were observed between the experimental and control groups, consistent with previous studies. Additionally, while the experimental group performed slightly better in flossing skills performance, the difference was not significant statistically. Achieving mastery in flossing skills requires repeated practice beyond a single session.

This study suggests combining theoretical learning with sustained practical efforts to achieve proficiency. Additionally, integrating strategies such as social support, habit formation, recognizing improvements, and enhancing health consciousness can further motivate learners and improve skill retention. In conclusion, the WSQ gamified learning approach shows promise for enhancing oral health education outcomes but requires refinement in motivational strategies and practical implementation for long-term impact.

Despite its benefits, the WSQ gamified learning approach faces some limitations. A key challenge is selecting a suitable gamified platform. Platforms capable of supporting large numbers of learners often involve high costs. The study recommends leveraging free platforms like *Gather*, which, despite limitations on simultaneous users, can facilitate group-based learning tasks. The WSQ gamified learning approach enables learners to engage effectively with the three stages of the WSQ strategy, thereby fostering higher-order thinking throughout the learning process. However, *Gather*’s interactivity is restricted to 2D gamified activities, limiting immersive 3D interactions. Collaboration with digital technology experts is recommended to develop a 3D educational gamified platform to better meet research and educational needs. Another limitation is the study’s small sample size and short experimental duration, which may lack sufficient statistical power to detect significant effects. Future research should include larger sample sizes and extending the study duration to validate these findings. Moreover, analyzing learners’ behaviors in gamified learning activities and incorporating group learning activities can provide deeper insights into the learning process and serve as a reference for further promoting gamified learning approaches.

## Data Availability

The data supporting the findings of the present study are available from the corresponding author upon reasonable request. The data supporting the findings of the present study are available from the corresponding author upon reasonable request.

## Abbreviations

WSQ: Watch-Summarize-Question
VR: Virtual reality
AR: Augmented reality
SCT: Social Cognitive Theory
UI: User interface
EVI: Expert Validity Index
